# Supplementary material for: Bacterial Proteases as Potentially Exploitable Modulators of SARS-CoV-2 Infection: Logic from the Literature, Informatics, and Inspiration from the Dog
Source: BioTech (Basel). 2023 Oct 30;12(4):61. doi: 10.3390/biotech12040061 (PMC10660736; doi:10.3390/biotech12040061)
Supplement: Supplementary file 1 [file biotech-12-00061-s001.zip › biotech-2527263-supplementary.pdf]

BioTech

Supplementary Information

Bacterial Proteases as Potentially Exploitable Modulators of SARS-CoV-2 Infection: Logic from Literature, Informatics, and Inspiration from the Dog

Gerald H. Lushington, Annika Linde and Tonatiuh Melgarejo

To support this hypothesis, several binding properties were computationally predicted for the SARS-CoV-2 Spike protein S2′ cleavage site associating with the proteolytic active site of human TMPRSS2, the Moraxella serine protease (MSP; GenBank ID: MBC7754020.1) and the Pseudomonas sp. serine protease (PSP; GenBank ID: MCL6711730.1). Specifically, of interest to probing the relative capacity of TMPRSS2, MSP and PSP to proteolytically prime the Spike protein to breach host cells, calculations were performed to predict **1)** the non-covalent binding free energy for a representative fragment of Spike binding to each of these protease active sites, **2)** the mean catalytically-relevant approach distances between the proteolytic serine oxygen and a proximal nucleophilic carbonyl carbon on the Spike backbone were assessed, and **3)** post-reaction simulations were performed to assess how readily the reaction products could dissociate from the protease.

These characteristics were simulated using the gaussian accelerated molecular dynamics (GAMD) [1,2] algorithm implemented in the NAMD [3] molecular modeling program. GAMD is an extensively validated model that, unlike conventional molecular dynamics simulations, employs more complex bonding, angle, torsion and non-bonding potentials that enable realistic sampling of a much greater conformational space that enhances free energy estimation, and essentially enables simulations of practical length (millions of time steps) to sample, discover and compare areas of biomolecular conformation space that might only be explored in a much greater number (comparable to greater than two orders of magnitude) of conventional simulation steps [1,2]. The GAMD protocol helps to alleviate conformational bias inherent in specifying the initial starting structure for a simulation. Although GAMD incurs greater computational expense per simulation step, the search versatility is of tremendous practical value in producing objective predictions for protease-substrate complexes that are still hypothetical (i.e., experimental proof of S2′ cleavage by MSP and PSP has not yet been reported), based on receptor structures (MSP and PSP) whose three dimensional structures can be computationally inferred through comparative modeling and threading, but whose precise native and complexed conformations have not been experimentally characterized.

For all simulations the viral substrate was modeled as a 27 amino acid peptide (P809 - K835) spanning the S2′ cleavage site (R815-S816) from the SARS-CoV-2 omicron BA.5 spike protein, as conformationally resolved in the crystal structure 7WEB [4], which was chosen as a reasonable constitutional representative of the Omicron subvariants (our targets at the time of analysis), with the structural manifestation of the S2 hairpin feature described as facilitating association between the Spike receptor-binding domain and ACE2 [5]. TMPRSS2 was modeled via the crystal structure 7MEQ [6]. The structure for MSP was solved for the Moraxella sp. serine protease sequence (GenBank ID MBC7754020.1), via default threading and 3D modeling functions within the Phyre2 server [7], with 3D structures resolved for residues 27-225. PSP was solved for the Pseudomonas sp. serine protease sequence (GenBank ID: WP_082643865.1) in the same manner as for MSP, with 3D structures resolved for residues 29-231.

Simulations to predict the catalytically-relevant substrate approach distances were prepared according to the prospective starting conformations delineated by Fig. S1. For each of these four prospective starting conformations, the substrate and protease were aligned by

hand in PyMol [8] by positioning the R815 backbone carbonyl carbon (sC) as closely as possible to the proteolytic serine side chain oxygen (pO) without incurring a substantial substrate-residue clash. Incremental constrained GAMD simulations were then performed. For each increment, the distance between sC and pO were rigidly constrained, all other degrees of freedom were subjected to 10000 steps of minimization, then were sequentially warmed (100 K for 20000 steps; 200 K for 20000 steps, 308 K for 1×10^5 steps) using NAMD (GAMD protocol; CHARMM 3.6 force field [9]; implicit solvent dielectric of $\varepsilon = 20.0$ via the GBSA model [10–12], as chosen to mimic a mucosal environment). At the end of the increment, the final structure was edited in PyMol to decrease the sC-pO distance by exactly 0.25 Å, and the aforementioned minimization and warming steps were repeated. Incremental approaches were continued until the sC-pO distance was less than 6.0 Å. At this point, each of the four different approach conformations for the Spike/TMPRSS2, Spike/MSP and Spike/PSP complexes was assessed for binding stability via an additional 1×10^6 steps at 308K with no constraints. For each substrate/receptor pair, the two conformations yielding the shortest mean sC-pO distance (over a 100 evenly sampled structures from the final 1×10^5 steps) were retained for further analysis.

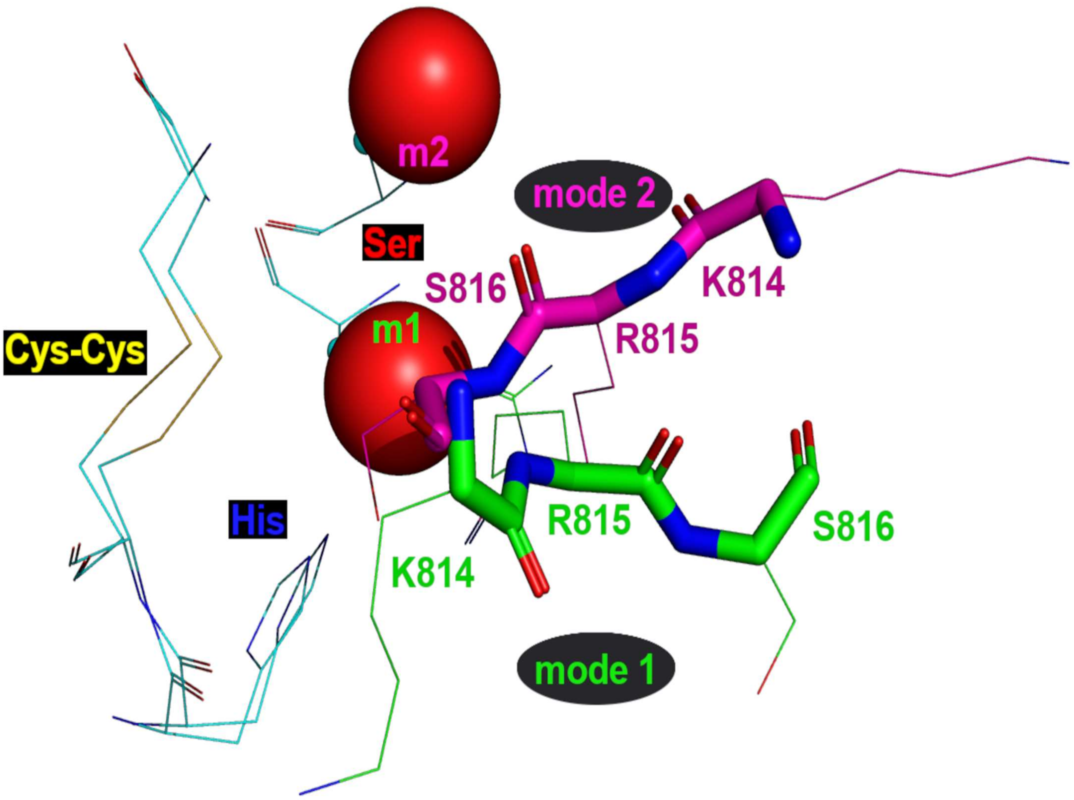

Figure S1. Orientations of S2′ coil bound to TMPRSS2-like proteases via either mode 1 (green) or mode 2 (magenta). For frame of reference key receptor features include the conserved disulfide bond (yellow), the amphoteric histidine (blue) and the catalytic / nucleophilic serine oxygen (red spheres). From receptor adaptive fit, we predict that the serine may adopts a different conformation depending on different substrate binding mode (m1 conformation for mode 1; m2 for mode 2).

Structures derived from the above protocol were used as a starting point for substrate approach distance analysis, and for binding free energy calculations. For each complex analyzed, the substrate approach assessment entailed profiling 1000 structures sampled evenly from the final 2×10^6 steps of a 5×10^6 GAMD simulation that propagate directly from the point of the preparative calculations described above. Free energy estimates were derived from these simulations via the standard Alchemical free energy perturbation in NAMD [3,13].

Prediction of the product release profiles was accomplished via two sequential simulations for each relevant complex. The first simulation entailed editing the final structure from the substrate approach simulation (as described above), such that a chemical bond was specified from the catalytic serine to the substrate carbonyl at the point of cleavage (suitable force field parameters specified using patches available in the CHARMM 3.6 parameter set), followed by manual cleavage (via PyMol; protons rearranged to yield normal valences) of the C-terminal fragment of the substrate. The resulting cleavaged structure was permitted to adapt to its altered state via a constrained incremental sC-pO adjustment, using the same strategy as described previously for construction of the non-covalent complexes, with the exception that incremental approach was halted once a sC-pO distance of less than 1.6 Å was achieved. Once a plausible sC-pO covalent distance had been attained in constrained form, the distance from the covalently bound sC atom to the manually dissociated amide nitrogen on the C-terminal leaving group was monitored over 1000 structures sampled evenly from a 5×10^6 GAMD simulation. Release of the N-terminal fragment was then simulated by taking the final structure from the C-terminal release simulation, manually editing the sC-pO bond to create a fully processed product, then optimizing the resulting structure (10,000 steps), followed by incremental unconstrained warming (100 K for 20000 steps; 200 K for 20000 steps, 308 K for 1×10^5 steps) followed by a full 5×10^6 GAMD simulation during which N-terminal fragment release was monitored as a function of unconstrained sC-pO distance.

References

1. Miao, Y; Feher, V.A.; McCammon, J.A. Gaussian Accelerated Molecular Dynamics: Unconstrained Enhanced Sampling and Free Energy Calculation. *J Chem Theory Comput* **2015**, *11*, 3584–3595.
2. Pang, Y.T.; Miao, Y.; Wang, Y.; McCammon, J.A. Gaussian Accelerated Molecular Dynamics in NAMD. *J Chem Theory Comput* **2017**, *13*, 9–19.
3. Phillips, J.C.; Hardy, D.J.; Maia, J.D.C.; Stone, J.E.; Ribeiro, J.V.; Bernardi, R.C.; Buch, R.; Fiorin, G.; Hénin, J.; Jiang, W.; McGreevy, R. et al. Scalable molecular dynamics on CPU and GPU architectures with NAMD. *J Chem Phys* **2020**, *153*, 044130.
4. Wang, K.; Jia, Z.; Bao, L.; Wang, L.; Cao, L.; Chi, H.; Hu, Y.; Li, Q.; Zhou, Y.; Jiang, Y. et al.; Memory B cell repertoire from triple vaccinees against diverse SARS-CoV-2 variants. *Nature* **2022**, *603*, 919–925.
5. Huang, Y.; Yang, C.; Xu, X.; Xu, W.; Liu, S. Structural and functional properties of SARS-CoV-2 spike protein: potential antivirus drug development for COVID-19. *Acta Pharmacol Sin* **2020**, *41*, 1141–1149.
6. Fraser, B.J.; Beldar, S.; Seitova, A.; Hutchinson, A.; Mannar, D.; Li, Y.; Kwon, D.; Tan, R.; Wilson, R.P.; Leopold, K. et al. Structure and activity of human TMPRSS2 protease implicated in SARS-CoV-2 activation. *Nat Chem Biol* **2022**, *18*, 963–971.
7. Kelley, L.A.; Mezulis, S.; Yates, C.M.; Wass, M.N.; Sternberg, M.J.E. The Phyre2 web portal for protein modeling, prediction and analysis. *Nat Protoc* **2015**, *10*, 845–858.
8. Lushington, G. Preface. *Comb. Chem. High. Throughput Screen*. **2021**, *24*, 1–2, DOI: 10.2174/138620732401210113110410
9. Huang, J.; MacKerell, A.D. CHARMM36 all-atom additive protein force field: validation based on comparison to NMR data. *J Comput Chem* **2013**, *34*, 2135–2145.
10. Im, W.; Feig, M.; Brooks, C.L. An Implicit Membrane Generalized Born Theory for the Study of Structure, Stability, and Interactions of Membrane Proteins. *Biophys J* **2003**, *85*, 2900–2918.
11. Onufriev, A.; Case, D.A.; Bashford, D. Effective Born radii in the generalized Born approximation: the importance of being perfect. *J Comput Chem* **2002**, *23*, 1297–1304.
12. Koehl, P. Electrostatics calculations: latest methodological advances. *Curr Opin Struct Biol* **2006**, *16*, 142–151.
13. Chipot, C.; Pohorille, A. (eds.) () *Free Energy Calculations: Theory and Applications in Chemistry and Biology* Springer, Berlin, Germany, 2007.
